# Preparation of nanocellulose and application of nanocellulose polyurethane composites

**DOI:** 10.1039/d4ra01412j

**Published:** 2024-06-07

**Authors:** Ya Mo, Xiaoyue Huang, Meng Yue, Lixin Hu, Chuanqun Hu

**Affiliations:** a School of Materials and Chemical Engineering, Hubei University of Technology Wuhan 430068 China moya0102@163.com huangxiaoyue0204@163.com yuemeng234@163.com Chem123@126.com hucqchem@mail.hbut.edu.cn

## Abstract

Polyurethane is a widely used material because of its excellent properties. Cellulose is a renewable, biocompatible, and biodegradable natural polymer that also has the advantages of a low density, high porosity, and large specific surface area. There are three main types of common nanocellulose: nanocellulose fibers, cellulose nanocrystals, and bacterial nanocellulose. Composites prepared with nanocellulose and polyurethane materials have good mechanical properties and good biocompatibility and can be applied in sensors, 3D printing, self-repairing materials, electromagnetic shielding, and many other areas. This paper details the preparation processes of different nanocelluloses and the application areas of composites, and points to the future development of nanocellulose polyurethane composites.

## Abstract 2

Types of nanocellulose and their application areas with polyurethane composites.

## Graphical abstract

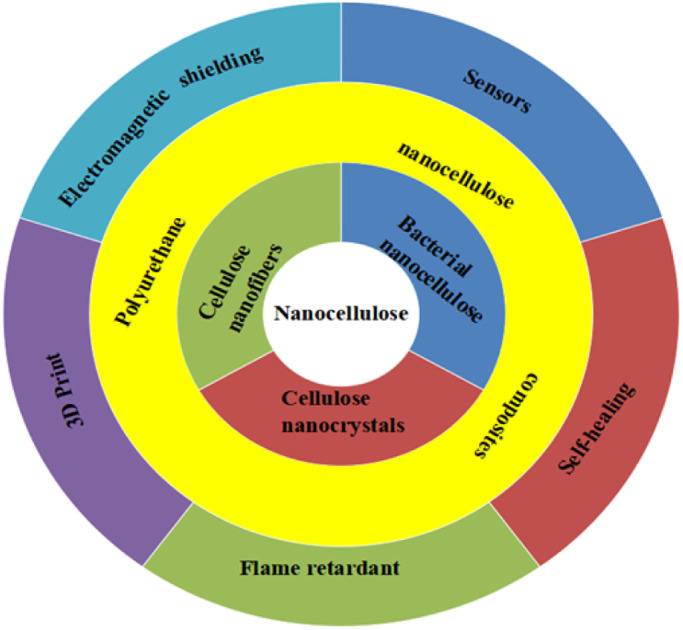

## 1. Introduction

Increasing production demands have accelerated the depletion of non-renewable resources such as oil. In order to meet the demand for resources, researchers are focusing on renewable natural polymer materials.

**Fig. 1:**
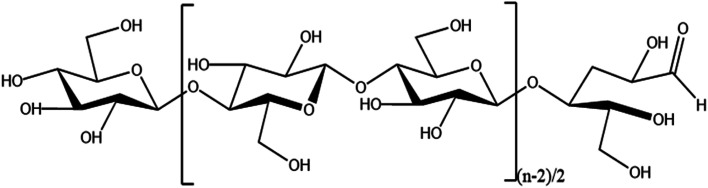
Structure of nanocellulose.

Cellulose is the most abundant natural polymer material in nature^1^ and is a linear polymer consisting of d-glucose linked to 1,4-β-glycosidic bonds.^2^ Cellulose has a supramolecular state structure; its solid state is represented by crystalline as well as amorphous regions,^3^ and it has many excellent characteristics such as biocompatibility, degradability, and thermal stability.^4,5^ Cellulose has multiple hydroxyl groups on each glucose molecule (as shown in Fig. 1), and cellulose can be esterified, oxidized, grafted, and more through these hydroxyl groups^6–8^ to modify it, which provides unlimited possibilities for the application of cellulose in the fields of biomedicine,^9^ energy materials,^10^ and fluorescent sensing.^11^

Polyurethane is a polymer made of polyisocyanate and polyhydroxy compounds and other polymers with a microphase-separated structure,^12^ which endows it with excellent mechanical properties,^13^ biocompatibility,^14^ abrasion resistance,^15^ and so on. These excellent properties ensure polyurethane materials have a wide range of applications in the fields of leather, 3D printing, biomedicine, sensors,^16–19^*etc.* Cellulose is amphiphilic, and its surface OH groups can combine with NCO groups in the polyurethane molecular chain, resulting in strong interfacial bonding. On the other hand, it can promote the dispersion of fillers in the polyurethane matrix.

## 2. Preparation of nanocellulose

Nanocellulose is defined as cellulose with a size of less than 100 nm in one dimension, and is mainly extracted from natural cellulose. Nanocellulose not only has many properties similar to cellulose itself, but also has the properties of nanomaterials, such as a high specific surface area, excellent mechanical properties, and thermal stability.^20^ There are three main types of common nanocellulose as cellulose nanofibers, cellulose nanocrystals, or bacterial nanocellulose.^21–23^

### 2.1 Cellulose nanofibers

Nanocellulose fiber is a cellulose-based nanomaterial with high strength, high modulus, low density, and excellent physical and chemical properties. Nanocellulose is obtained through nanofabrication of natural cellulose and exhibits a unique structure and properties distinct from those of conventional cellulose. It has a high length-to-diameter ratio and exhibits a nanowire-like microstructure. The diameter of the nanocellulose fiber is typically between 100 and 1000 nanometers, with lengths reaching the micrometer level. Moreover, it is distinguished by its high strength, high modulus, low density, excellent physical properties, and biodegradability. Cellulose nanofibers are mainly prepared by mechanical methods (cutting cellulose fibers into nanoscale lengths by mechanical grinding and stretching), chemical methods (modifying cellulose using catalysts such as acids, bases or enzymes, and then preparing nanofibers through steps such as filtration, washing and drying), and biological methods (using microbial fermentation or fungal degradation of cellulose to produce nanofibers).

**Fig. 2:**
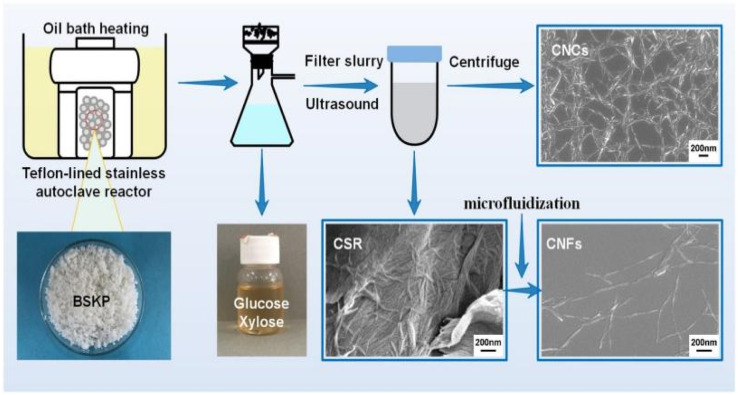
Nanocellulose was prepared by hydrolyzing with dilute sulfuric acid and mechanical fibrination.^25^

Fukugaichi *et al.*^24^ prepared lignocellulose nanofibers (LCNFs) from plantain pseudostems *via* a simple one-step NaOH treatment. This treatment removed hemicellulose and destroyed the lignin present in cellulose's microprimary fibers while breaking the hydrogen bonds therein to extract the LCNFs. The extracted LCNFs had high aspect ratios, they were heat resistant up to 352 °C, and they were predominantly type I cellulose, with a content of up to 71%. Wang *et al.*^25^ prepared cellulose nanocrystals and cellulose nanofibers simultaneously *via* hydrolysis with dilute sulfuric acid and mechanical fibrillation under hydrothermal heat using incompletely hydrolyzed cellulose residues as the raw material (Fig. 2). The sulfuric acid concentration used in this method was only 0.3%, and the resulting cellulose nanofibers had a high aspect ratio. Yields of up to 69% were achieved with a maximum degradation rate temperature of 350 °C. This process provides a clean method for manufacturing biomass nanomaterials. Chen *et al.*^26^ prepared cellulose nanofibers (P-CNFs) with natural antimicrobial properties by hydrolyzing pinecones using H_2_O_2_ at 60 °C. The P-CNFs had a high thermal decomposition temperature and can be easily dispersed in ethanol. The minimum inhibitory concentrations (MICs) for *E. coli* and *S. aureus* were as low as 1.5 mg ml^−1^ and 2 mg ml^−1^, respectively. P-CNFs can be combined with 75% ethanol to form a disinfectant that effectively inhibits bacterial growth. Wang *et al.*^27^ produced cellulose nanofibers from sugarcane bagasse using a synergistic ternary mechanism comprising oxidation, swelling, and acid hydrolysis under mild conditions. The resulting CNFs had a width of 12 nm, had a crystallinity of 64%, and exhibited a good thermal stability. The CNF yield varied depending on the initial cellulose content in the biomass and the initial biomass content, with yields reaching up to 93% and 50%, respectively. Lu *et al.*^28^ produced fluorescent cellulose nanofibers (FCNFs) with high yields through one-pot hydrolysis and thiazolopyridine carboxylic acid (TPCA) grafting. Under microwave hydrothermal conditions, cellulose undergoes hydrolysis and Fischer esterification, resulting in a 73.2% yield of FCNFs at a microwave power of 500 W, a reaction temperature of 110 °C, and a reaction time of 5 h. The FCNFs were prepared in the form of short rods and had a crystallinity of 80%. Furthermore, their thermal stability remained unchanged. Additionally, the FCNFs exhibited exceptional fluorescence properties and they were highly responsive to chloride ions.

### 2.2 Cellulose nanocrystals

Cellulose nanocrystals are a type of nanoscale crystalline material derived from natural cellulose. These materials possess several advantageous properties, including renewability, biodegradability, non-toxicity, and high strength. Cellulose nanocrystals exhibit a highly ordered crystal structure, with a diameter generally between 10 and 50 nanometers and a length of up to hundreds of nanometers. Due to their unique crystal structure, cellulose nanocrystals exhibit high tensile strength and modulus. Cellulose nanocrystals are mainly prepared by mechanical methods (obtaining nano-sized cellulose crystals by processing fiber raw materials through high-pressure homogenization or mechanical ball milling), chemical methods (preparing cellulose materials into nanoparticles through chemical reactions), biological methods (converting cellulose raw materials into cellulose nanocrystals through the use of microbial fermentation or enzymolysis), and solvent methods (solvent method, which is prepared by dissolving cellulose in suitable solvents. Cellulose nanocrystals by dissolving cellulose in a suitable solvent, and then preparing cellulose nanocrystals through evaporation, drying and other processes).

**Fig. 3:**
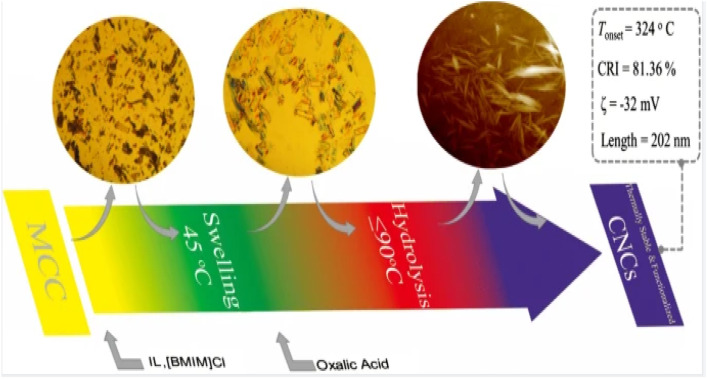
Preparation of cellulose nanocrystals by ionic liquid (IL)-mediated technique.^29^

Fu *et al.*^29^ produced thermally stable and surface-functionalized cellulose nanocrystals (CNCs) using fully recyclable organic acids and ionic liquid (IL)-mediated techniques under mild conditions. To achieve this, they first pretreated microcrystalline cellulose with an IL-controlled portion. Then, they used an organic acid (oxalic acid) to hydrolyze the MCC cellulose chains in the amorphous region, resulting in the production of surface-functionalized CNCs at less than 100 °C. The method used led to CNCs with an average length of 202 nm, a good thermal stability (with an initial decomposition temperature of 324 °C), a high degree of crystallinity of 81.36% (Fig. 3), and a high surface charge loading. Wang *et al.*^30^ proposed a sustainable and efficient method to prepare bifunctional cellulose nanocrystals (CNCs) from bleached eucalyptus kraft pulp (BEKP) using a mixed system of sulfuric acid and formic acid (FA). Using this method can enhance the formic acid (FA) hydrolysis rate in the presence of a small amount of sulfuric acid to efficiently produce CNCs. A yield of up to 70.65% was achieved. The prepared CNCs exhibited a better thermal stability (with an initial decomposition temperature of up to 288 °C), a high crystallinity, and good water dispersion compared to the CNCs prepared *via* the conventional acid digestion method. Wang *et al.*^31^ utilized an acid hydrolysis system to break down cellulose pulp into rod-shaped CNCs. The system consisted of small amounts of sulfuric acid (5–10%) and large amounts of easily recoverable acetic acid (70–90%). The raw material was bleached eucalyptus kraft pulp, and the process was carried out at 80 °C for several hours. The obtained CNCs had lengths ranging from 150 to 500 nm and diameters of 5–20 nm, with yields of up to 81%. The results indicate that the CNCs produced through this method exhibit a high thermal stability and good dispersion in both aqueous and organic phases. Zhou *et al.*^32^ prepared cellulose nanocrystals using a synergistic process of sulfuric acid hydrolysis and the hot-wet method. They hydrolyzed microcrystalline cellulose at 100 °C using low amounts of diluted sulfuric acid (0.5 M or 1 M) due to its high reaction rate. The CNCs produced *via* this method were rod-like (100 nm) and spherical (10 nm) in shape, with a relative crystallinity ranging from 70.92% to 81.13%, an average particle size of 320 nm, and a yield of up to 93.68%. Worku *et al.*^33^ prepared carboxylated nanocrystals (CNCs) from oxidized sphagnum cellulose using anhydrous citric acid (85–100 wt%) and a small amount of sulfuric acid (0–15 wt%) as catalysts. The highest yield of CNCs, 89.7%, was achieved at an anhydrous citric acid to sulfuric acid ratio of 9 : 1 wt%, a reaction temperature of 80 °C, and a reaction time of 5 h. The test results indicate that the CNC particle size was 68.06 ± 1.05 nm, the maximum absolute zeta potential was −33 mV, and the crystallinity ranged from 60.37% to 81.3%.

### 2.3 Bacterial nanocellulose

Bacterial nanocellulose is a nanoscale cellulose material produced by bacteria. In contrast to conventional sources of cellulose, bacterial nanocellulose is produced through a bacterial fermentation process, resulting in a material with high purity, high crystallinity, and a unique nanostructure. Bacterial nanocellulose can be prepared by microbial fermentation (using specific bacteria to ferment cellulose raw materials and convert cellulose into nanocellulose), enzymatic digestion (using cellulase to enzymatically digest cellulose raw materials and break down the cellulose into nanocellulose), and microbial fermentation-enzymatic digestion (combining the two methods of microbial fermentation and enzymatic digestion, the cellulose raw materials are firstly converted into more easy to be enzymatically digested through fermentation, and then enzymatically digested using cellulase to prepare nanocellulose). (combining the two methods of microbial fermentation and enzymatic digestion, the cellulose raw material is first converted into a more easily digestible substance through fermentation, and then cellulase is used for enzymatic digestion to prepare nanocellulose).

**Fig. 4:**
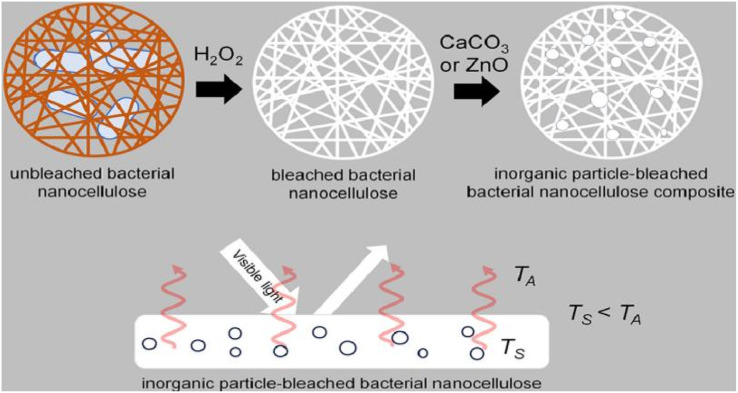
Preparation of bacterial nanocellulose by Kombucha fermentation.^34^

Machfidho *et al.*^34^ produced bacterial nanofibrillar cellulose *via* kombucha fermentation (Fig. 4). The resulting bacterial nanocellulose was then bleached with hydrogen peroxide to remove impurities. The bleached bacterial nanocellulose exhibited a nanofibrous shape and a cellulose I crystalline structure with 44.3% crystallinity. The fiber diameter was approximately 92.51 ± 21.46 nm. Sijabat *et al.*^35^ synthesized bacterial nanocellulose (BNC) from banana peel waste using the process of *Bacillus xylosus* fermentation. This was achieved at a pH of 4, with 0.5% urea and sucrose contents of 5%, 10%, and 15% (w/v). The thickness of the BNC increased proportionally with the sucrose content. The method produced nanofibers of 30–50 nm in size. Nyakuma *et al.*^36^ synthesized bacterial nanofibrillar cellulose (BNC) using acinetobacter glucose. The prepared BNC was studied *via* thermogravimetric analysis, scanning electron microscopy, and kinetic modeling. This study revealed that BNC possesses a dense fibrous structure with overlapping nodes, indicating a high specific surface area, porosity, and crystallinity. Kinetic modeling demonstrated that BNC has a high thermo-reactive activity, as evidenced by its average activation energy (*E*_a_ = 59.39 kJ mol^−1^) and exponential pre-factor (*k*_o_ = 1.62 × 10^10^ min^−1^) (Table 1).

**Table 1:** Results of preparation of nanocellulose

Type of study carried out	Results	Reference
This work used a one-step NaOH treatment to prepare lignocellulosic nanofibers	Heat-resistant temperature of up to 352 °C; main component: type I cellulose; yield of up to 71%	24
In this work, cellulose nanocrystals and cellulose nanofibers were prepared simultaneously *via* hydrothermal hydrolysis of dilute sulfuric acid and mechanical fibrillation under water heat	Yields up to 69% with a maximum degradation rate temperature of 350 °C	25
This work hydrolyzed pinecones to prepare a cellulose nanofiber with natural antimicrobial properties	Crystallinity increased from 64.93 to 67.75%	26
This work prepared cellulose nanofibers from sugarcane bagasse by using phosphoric acid and hydrogen peroxide in the one-bath method	CNFs width of about 12 nm, yields (93% and 50% based on cellulose and biomass, respectively), a crystallinity of 64%	27
In this work, fluorescent cellulose nanofibers were prepared in a high yield using one-pot hydrolysis and the thiazolopyridine carboxylic acid (TPCA) grafting method	The yield of FCNFs was 73.2%, and the prepared FCNFs were in the form of short rods. Crystallinity was 80%	28
This work used fully recyclable organic acids and ionic liquid (IL)-mediated techniques to prepare thermally stable and surface-functionalized cellulose nanocrystals (CNCs)	CNC length was 202 nm, the initial decomposition temperature was 324 °C, crystallinity was 81.36%	29
This work prepared cellulose nanocrystals from bleached eucalyptus kraft pulp using a mixed acid system of sulfuric acid and formic acid (FA)	Yields was 70.65%, an initial decomposition temperature of up to 288 °C	30
In this work, cellulose nanocrystals were prepared from bleached eucalyptus kraft pulp by using an acid hydrolysis system consisting of sulfuric and acetic acids	CNCs lengths of 150–500 nm and diameters of 5–20 nm, yields was 81%	31
This work prepared cellulose nanocrystals *via* a synergistic process of sulfuric acid hydrolysis and the hot-wet process	The CNCs rod-like (100 nm), and spherical (10 nm),crystallinity of 70.92–81.13%, an average particle size of 320 nm, yield was 93.68%	32
Preparation of carboxylated nanocrystals from oxidized stilbene cellulose using anhydrous citric acid and a small amount of sulfuric acid as catalysts	CNC particle size was 68.06 ± 1.05 nm, the maximum absolute zeta potential was −33 mV, crystallinity 81.3%, the yield was 89.7%	33
In this work, bacterial nanofibrillar cellulose was prepared *via* the Kampo tea fermentation method	The crystallinity was 44.3%. The fiber diameter was about 92.51 ± 21.46 nm	34
This work synthesized bacterial nanocellulose from banana peel waste using a *Bacillus xylococcus* fermentation process	The bacterial nanofibrillar cellulose consisted of nanofibers of 30–50 nm in size	35
This work synthesized bacterial nanofibrillar cellulose using acinetobacter glucose	BNC kinetic modeling showed that the average activation energy was *E*_a_ = 59.39 kJ mol^−1^. The exponential prefactor *k*_o_ = 1.62 × 10^10^ min^−1^	36

## 3. Nanocellulose/polyurethane composite applications

The hydroxyl group on the surface of cellulose can produce strong interfacial interactions with the polyurethane molecular chain through hydrogen bonding, which improves the mechanical properties and the thermal stability^37,38^ of the composites. On the other hand, cellulose is amphiphilic and can promote the dispersion of fillers in the polyurethane matrix. Nanocellulose/polyurethane composites have a wide range of applications in the fields of sensing, 3D printing, self-repairing materials, electromagnetic shielding, and flame retardants.^39–43^

### 3.1 Sensors

Nanocellulose is amphiphilic, and this can promote the dispersion of conductive fillers in the matrix, improve sensitivity by forming a brittle conductive network, and also improve the mechanical properties of the matrix.

**Fig. 5:**
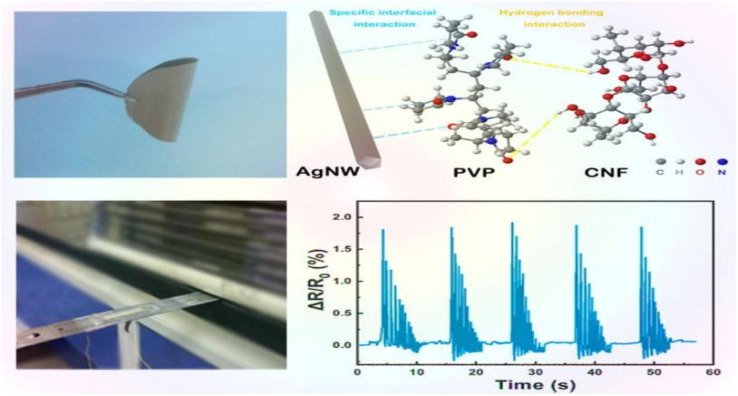
AgNW/CNF hybrid nanopaper and real-time variation in the resistance of the bending sensor upon external damp vibrations.^46^

Zhai *et al.*^44^ prepared composite aerogels consisting of carbon nanotubes, graphene, waterborne polyurethane, and cellulose nanocrystals (CNTs/graphene/WC) using a readily soluble solution mixing and freeze-drying technique. These aerogels were used to create high-performance pressure sensors. The pressure sensors that were prepared exhibit a highly porous network structure, excellent mechanical properties (76.16 kPa), a high sensitivity (0.25 kPa(−1)), an ultra-low detection limit (0.112 kPa), and a high stability (>800 cycles). These characteristics highlight their promise for use in flexible and wearable electronic products. Zhang *et al.*^45^ utilized the amphiphilic nature of cellulose nanocrystals (CNCs) to design a high-compression aqueous polyurethane/carbon nanotube composite foam material with enhanced piezoresistive properties. The sensitivity of this foam increased 2.5-fold compared to the foam without CNCs. It had good compressibility and stable piezoresistive sensing signals in an 80% compressive strain range with a response time of 30 ms. The foam still possessed a good sensing performance after 1000 cycle tests. Yin *et al.*^46^ prepared composite nanopaper of silver nanowires (AgNWs) and cellulose nanofibers (CNFs) using face-liquid mixing and vacuum filtration techniques. They sandwiched the nanopaper between two layers of thermoplastic polyurethane (TPU) film using heat compression to create a tensile strain sensor. Additionally, they constructed a special microcracked structure through a pre-strain process to enhance sensitivity. The test results indicated that the sensor had a low detection limit of 0.2% and exhibited excellent stability and durability after 500 cycles (Fig. 5).Yan *et al.*^47^ prepared a self-healing polyurethane sponge reinforced with biopolymers that contains reversible oxime–carbamate bonds. Based on this, they developed a lightweight piezoresistive sensor using silver nanoparticles, carbon nanotubes, cellulose nanocrystals, and tannic-acid-modified polyurethane (AgNPs/CNTs-CNCs@TA-PU) sponges through a simple iterative dip-drying process. The sensor has a wide compressive stress range (0–788.3 kPa) and excellent sensitivity and durability. It can self-heal by heating at 110 °C for 1 hour with an efficiency of 80.3%. Its GF is as high as 17.1 in the 0–1% compressive strain range. Chen *et al.*^48^ developed a piezoresistive sensor by using a composite coating of carbon black (CB), cellulose nanofibers (CNFs), thermoplastic polyurethane (TPU), and thermoplastic elastomer styrene-ethylene/butylene-styrene (SEBS). The test results indicated that the sensor had a sensitivity of 0.0316 kPa^−1^, a detection range of over 200 kPa, a cycle count of over 1000, and a response time of 0.5 ms. Zhang *et al.*^49^ developed a piezoresistive sensor by dip-coating a ruptured cellulose nanofiber/silver nanowire (CA) layer onto a polyurethane (PU) sponge using a simple dip-coating process and pre-compression treatment. The sensor can detect both large and small motion across a wide compressive strain range of 0–80%. It has a low quality detection limit of 0.2% and a GF of up to 26.07 over a strain range of 0–0.6%. This sensor demonstrated an excellent stability, repeatability, and durability over 500 cycles. Xu *et al.*^50^ developed a 3D piezoelectric sensor by coating a conductive layer of CNF@CB onto a polyurethane foam substrate after low-pressure oxygen plasma treatment. The sensor exhibited a sensitivity of up to 0.35 kPa(−1), a compressive force of up to 29 kPa at 50% stress, and a conductivity of 0.047 S m^−1^. Additionally, the sensor demonstrated excellent cycling performance and stability over 1000 cycles. Li *et al.*^51^ developed a flexible conductive sensor by dip-coating a MXene/cellulose nanocrystalline-coated thermoplastic polyurethane nonwoven fabric. The conductive nonwoven fabric was pre-stretched, resulting in a sensor with a wide sensing range of 83%, a high sensitivity (GF = 3405), and an ultra-low detection limit (0.1%) based on the opening and closing of microcracks. Cui *et al.*^52^ developed a strain sensor composed of a conductive layer of cellulose nanocrystals (CNCs), carbon nanotubes (CNTs), and a MXene nanohybrid network, and a stretchable elastomer layer made of thermoplastic polyurethane and a fluorophore. The sensor exhibited an ultra-high sensitivity (476 800), a low detection limit of 0.005%, a response time of 60 ms, and a wide response range of 0–100%. Miao *et al.*^53^ prepared a monodisperse mixed aqueous slurry of carbon nanotubes (CNTs) and MXene to formulate a composite ink with aqueous thermoplastic polyurethane and nanocellulose. Even with a low content of 0.08% CNTs and MXene in the ink, a stable sensing conductive network with a conductivity of 127 s cm^−1^ was formed. The ink sensor exhibited a high sensitivity of 43.61 in the tensile test, and the sensor resistance changed linearly under 0–89% strain conditions. Additionally, the sensor resistance change remained stable after 2100 fatigue tests.

### 3.2 Three-dimensional printing

Three-dimensional printing can be used to prepare functional materials, offering advantages such as structural customization, which can promote the development of functional materials and has huge application potential. At present, due to the limitations of molding technology, 3D printing of polymers and composites is extremely limited. The viscosity of polymers in solution is adjustable, and the solution has shear thinning characteristics, providing the necessary conditions for the 3D printing process.

**Fig. 6:**
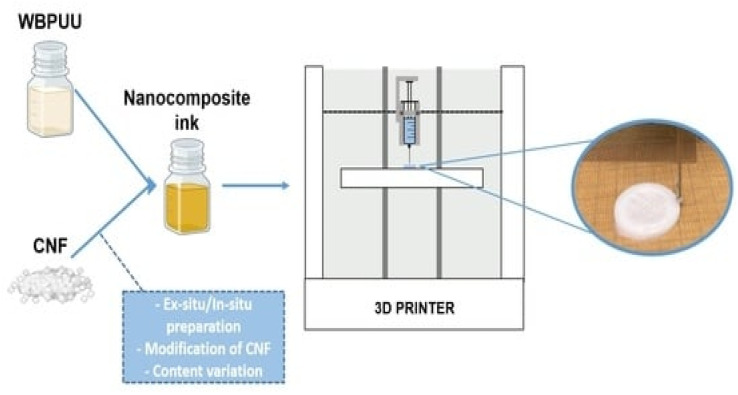
Three-dimensional-printed waterborne polyurethane-urea-based nanocomposite inks.^56^

Zhou *et al.*^54^ utilized a 3D printing molding technique to incorporate cellulose and nanocellulose into SMPUs, enhancing the overall performance of the porous SMPUs. The solidity (*R*_f_) and shape recovery (*R*_r_) of cellulose were increased to over 99.0% by adding the appropriate proportions of cellulose nanocrystals (CNCs) and ball-milled nanocellulose (BMC). The maximum tensile strength of the 3D-printed SMPU composites was 12.05 MPa, which is 71% higher than that of pure PU. These composites have a tunable glass transition temperature, and composites with *T*_g_ temperatures close to the human body temperature can be obtained under appropriate preparation conditions. Chen *et al.*^55^ used cellulose nanofibrils (CNFs) to prepare printable PU composites. The viscosity of the composites was effectively regulated by the neutralizer amount during *in situ* synthesis. The resulting 3D-printed PU/CNF scaffolds exhibited excellent pattern fidelity and structural stability. Meanwhile, the compression modulus of the PU/CNF scaffolds was higher than that of the scaffolds printed with the water-soluble viscosity-enhancer PEO. Additionally, the cell proliferation rate of the PU/CNF scaffolds was faster than that of the PU/PEO scaffolds. After 14 days, the number of viable cells in the PU/CNF scaffolds was approximately five times (537%) the initial number. Larraza *et al.*^56^ prepared waterborne polyurethane-urea (WBPUU)-based inks using both non-*in situ* and *in situ* doping methods (Fig. 6). Modified CNFs were also employed to improve the affinity with the substrate. In the non-*in situ* preparation, the interaction between the CNFs and water was superior to that between the CNFs and the WBPUU nanoparticles, resulting in a strong gelatinous structure. The more robust gel-like behavior resulted in more precise 3D-printed parts, thereby improving their mechanical properties. Kothavade *et al.*^57^ increased the toughness of polylactic acid (PLA) by 223% and the tensile modulus by 21% by incorporating pyrene butyric-acid-modified cellulose nanofibers (PBA-m-CNFs) and thermoplastic polyurethane (TPU) at a concentration of 10%. The fracture cross-section of the 3D-printed composites exhibited a ductile damage mode. Additionally, the solid-state fluorescence quantum yield of 3D-printed PLA/PBA-m-CNF1/TPU10 composites was as high as 38.35%.

### 3.3 Self-healing materials

A self-repairing function can be introduced by generally one of two ways: one way is to introduce reversible bonds into the polymer network; if the material is damaged, it can directly respond. The other way is to join the carrier with a repair agent in the material; if the material has crack damage, the repair agent flows to the crack to self-repair. The mechanical properties of self-repairing materials with reversible bonds are often very poor; nanocellulose is often used as filler for composites to improve the performance of composites due to its good thermal stability and high crystallinity.

**Fig. 7:**
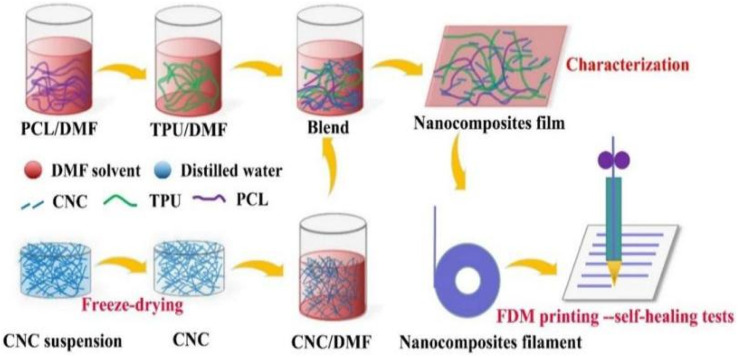
TPU/PCL/CNC nanocomposite preparation and FDM printing process.^60^

Fan *et al.*^58^ developed self-repairable supramolecular composites at room temperature using polyurethane elastomers as a matrix, incorporating cellulose nanocrystals (CNC) and multiple dynamic bonds. A dynamic cross-linking network was generated through hydrogen bond formation between the hydroxyl groups on the surface of the CNC and the matrix, enabling the composite to self-repair without compromising its mechanical properties. The composites exhibit favorable mechanical properties, with a self-repair rate of up to approximately 95%. This makes them highly promising for use in flexible electronic devices. Bi *et al.*^59^ created a thermally reversible crosslinked network by modifying cellulose nanocrystals with thermoplastic polyurethane (TPU) and polycaprolactone (PCL). They used furan groups and maleic anhydride as the crosslinking agents. The composites were enhanced in terms of their mechanical properties, thermal stability, and self-healing properties due to the physical and chemical cross-linking structures formed by the modified CNC. The composites' fracture strength and elongation could be restored to 87% and 91%, respectively, through self-repair. Bi *et al.*,^60^ prepared a polymer blend using a solution casting method (Fig. 7). The blend consisted of thermoplastic polyurethane and polycaprolactone. Cellulose nanocrystals were introduced to enhance interfacial compatibility using 3D printing techniques. The blends that contained 1% CNCs exhibited excellent mechanical properties. Specifically, they had a tensile strength of 31 MPa and an elongation at break of 1600%. Additionally, they maintained good self-healing properties even after three fractures, and their mechanical strength was as high as 83.5% of that of the raw material after self-healing. Yang *et al.*^61^ developed a new amorphous transparent polyurethane/nanocellulose elastomer with exceptional self-healing, self-reinforcing, and toughening abilities. They achieved this by implementing a bi-dynamic cross-linking strategy that utilized multiple hydrogen and disulfide bonds. Hydrogen bonds were introduced in tempo oxidized cellulose nanofibers (TCNFs) and disulfide bonds (SS) were introduced into the polyurethane (PU) backbone to form a bi-dynamic crosslinked network. As a result, the tensile strength and toughness of the elastomer samples increased by 401% and 257%, respectively, compared to the pristine samples. Liu *et al.*^62^ prepared self-healing waterborne polyurethane (WPU) coatings with a high transparency and haze using cellulose nanocrystal (CNC)-stabilized flaxseed oil (LO) skinned emulsion. They investigated the self-healing properties of the coatings under varying conditions of time, temperature, CNC content, and catalyst concentration. The results indicate that CNC incorporation improved the abrasion resistance and mechanical properties of WPU coatings compared to pristine WPUs, and also resulted in good self-healing properties. Additionally, the coatings demonstrated a high transparency and a low haze.

### 3.4 Electromagnetic shielding

The preparation of electromagnetic shielding materials by combining cellulose with metals or metal oxides, carbon materials, polymers, or MXene is based on electromagnetic wave loss theory.

**Fig. 8:**
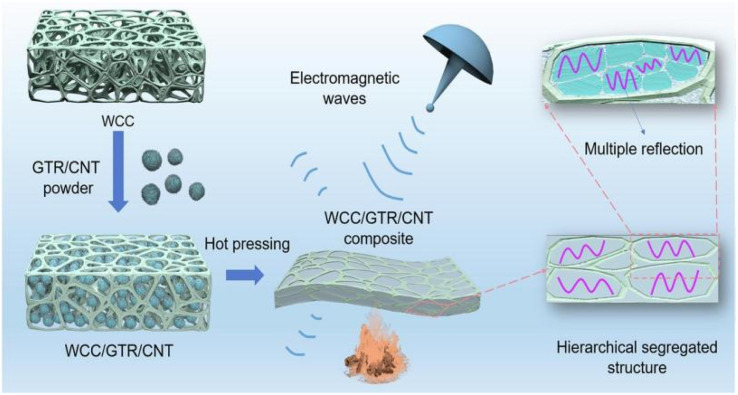
Schematic diagram of the preparation of WCC/GTR/CNT composites.^66^

Zhao *et al.*^63^ prepared bilayer-structured composite MXene/CNC/WPU (MCW-X) films through vacuum-assisted alternating filtration. The electrical conductivity and stability of the MCW-X material were optimized *via* the formation of a conductive network between CNC and MXene. Additionally, this enhanced the mechanical properties of MXene nanosheets through interactions between WPU and MXene. Meanwhile, the inclusion of CNC and WPU into MXene nanosheets expanded the interlayer spaces in the composite film, optimizing impedance matching and enhancing the electromagnetic shielding performance. The resulting film exhibited a high-performance EMI shielding of 54.2 dB in the X-band. Shang *et al.*^64^ prepared sandwich films consisting of cellulose nanofibers, boron nitride nanosheets, and Ti_3_C_2_T_*x*_ MXene using a simple alternating vacuum filtration method. This unique sandwich structure resulted in films with outstanding electromagnetic shielding, insulation, and thermal conductivity properties. The composite film exhibited a high electromagnetic shielding effectiveness of 60.7 dB at 8.2 GHz while maintaining good electrical insulation properties. This is due to the presence of a CNF/BNNS layer on the surface. Zhang *et al.*^65^ transformed waste polyurethane foam (WPUF) into a high-performance electromagnetic interference (EMI) shielding material by surface-coating carbon nanotubes and applying a thermo-compression treatment. The intact three-dimensional skeleton of the WPUF's novel skin-core separation structure was well preserved after compression molding. The polyurethane skeleton's surface features uniformly distributed multi-walled carbon nanotubes (MWCNTs) supported by cellulose nanofibers (CNFs). This distribution provides the material with a remarkable electrical conductivity, which leads to an excellent EMI shielding performance (up to 63.2 dB). Zhang *et al.*^66^ investigated an electromagnetic shielding material with a strong conductive skeleton similar to that of the ‘rebar’ in reinforced concrete. They formed a ‘concrete’-like composite of waste flame retardant polyurethane foam (WFPUF), ground tire rubber (GTR), carbon nanotubes (CNTs), and cellulose nanofibers (CNFs). Separate structures of GTR and CNTs made up the WCC/GTR/CNT composite (Fig. 8). This results in the composites having excellent mechanical properties, electrical conductivities, and electromagnetic shielding properties (up to 53.8 dB). Wang *et al.*^67^ created MXene composite aerogels by co-assembling MXene and cellulose nanofibers during freeze-drying. They then encapsulated the surface with flame-retardant thermoplastic polyurethane (TPU). The aerogel fully utilizes the pore structure of MXene and enhances conductive loss through multiple internal reflections. Its electromagnetic shielding performance can reach 93.5 dB. Furthermore, the aerogel exhibits superelasticity due to multiple hydrogen bonds and the presence of TPU elastomers. Additionally, it possesses exceptional properties, including an increased strength and stability. Kong *et al.*^68^ utilized WPU to combine absorbing monomeric CNT/CNF in a layered structure. They obtained an elastic CNT/CNF-WPU layered composite aerogel through a simple bidirectional lyophilization method. The CNT/CNF content in the CNT/CNF-WPU aerogel was 2.5 wt%, resulting in a minimum reflection loss (RL_min_) of −77.3 dB and an effective absorption bandwidth (EAB) that covered the entire X-band. A sample with a thickness of 4.9 mm can achieve an RL of less than −13.4 dB across the full X-band frequency. The CNT/CNF-WPU aerogel exhibits a directionally aligned lamellar structure, providing very stable EMW absorption performance. Additionally, it can be recycled up to 100 times at 60% strain.

### 3.5 Flame retardant

The polyhydroxy structure of cellulose makes chemical modification a major way to improve flame retardancy properties. It has a large specific surface area, which is conducive to the formation of a dense protective layer on the surface of the material, thus slowing down the combustion process. Secondly, nanocellulose has a high thermal stability, meaning the structure is stable at high temperatures, thus slowing down material combustion. In addition, nanocellulose forms a protective carbon layer during the combustion process, which further prevents the spread of flames.

**Fig. 9:**
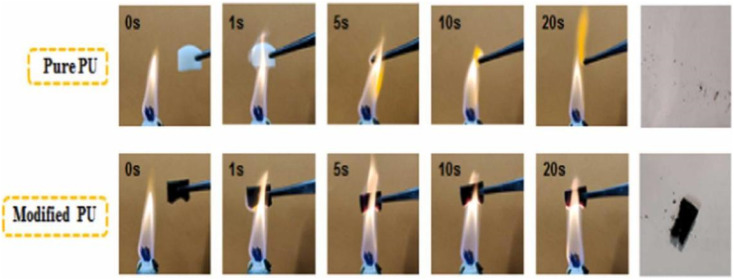
Combustion process of a PU sponge.^69^

Huang *et al.*^69^ produced biomass particles consisting of hydrothermally treated lignin nanoparticles (HLNPs)/Fe_3_O_4_ through solvent displacement and a hydrothermal reaction. They then used cellulose nanocrystals (CNCs) and TiO_2_ as reinforcements and PDMS as a binder to create a superhydrophobic polyurethane coating with flame retardant properties (Fig. 9). The resulting polyurethane sponge exhibited a hydrophobicity angle of up to 167°, withstanding 87 anti-abrasion cycles, and had a limiting oxygen index (LOI) of 27.3%. Kim *et al.*,^70^ incorporated acylated nanocellulose into a polyurethane matrix containing the conventional flame-retardant tris(2-chloroethyl) phosphate (TCEP). This resulted in the faster production of char layers during combustion, leading to delayed flame propagation. The samples showed an increased limiting oxygen index (LOI) of 29% and a significant reduction in toxic emissions production. During the cone calorimetry test, the samples exhibited a low heat release rate (HRR) and smoke production rate (SPR), indicating a high flame retardancy and a relatively low environmental impact. Kim *et al.*^71^ conducted a study to reduce smoke emission during combustion by adding silylated and nano-fibrillated cellulose (Si-NFC) to polyurethane foam (PUF) containing tris(2-chloropropyl) phosphoric acid (TCPP). The thermal properties of the composite foam were investigated *via* thermogravimetric analysis and limiting oxygen index (LOI) and cone calorimeter tests. The Si-NFC-embedded composite exhibited an increase in the LOI from 19.3% to 24.6%. Furthermore, the use of Si-NFC resulted in an enhancement in the composite's thermal stability, as evidenced by a decrease in the peak release of heat and smoke. Luo *et al.*^72^ improved the flame retardancy of polyurethane foam by microencapsulating ammonium polyphosphate (APP) with nanocellulose and dicyandiamide-formaldehyde using *in situ* polymerization and flocculation. They then prepared flame-retardant materials by combining microencapsulated ammonium polyphosphate (DFNAPP) with polyurethane foam. The test results showed that the LOI of the flame-retardant material increased by 22.1% compared to APP, and the compressive strength of the material increased from 195 kPa to 213 kPa.

## 4. Conclusions

Cellulose, as the most widely available polymer material on earth, is a significant candidate to replace non-renewable materials. Nanocellulose is an appropriate choice to improve the properties of composite materials due to its good biocompatibility and mechanical properties. Polyurethane materials have good mechanical and biocompatible properties due to their microphase separation structure, and the preparation of flexible electronic devices *via* the combination of polyurethane materials and nanocellulose has become a hot spot in current research.

There are still some problems in the preparation and application of nanocellulose–polyurethane composites. For example, (1) the cost of nanocellulose–polyurethane composite production is high, the aforementioned factors are not conducive to the implementation of industrialized development applications. In addition, using polyurethane and nanocellulose in the production process requires the use of organic solvents and organic catalysts, which have a certain impact on the human body and the environment. (2) The oxidation resistance of nanocellulose polyurethane composites needs to be improved, some components of composites are susceptible to the generation of free radicals in oxidizing environments leading to degradation of material properties.

Therefore, future research and development of nanocellulose polyurethane composites must combat the problems of environmental pollution, high cost, and low oxidation resistance. To increase nanocellulose utilization, it is necessary to explore efficient, environmentally friendly, and low-cost production processes, while all biobased non-isocyanate polyurethanes should be studied in depth to discover alternatives to traditional polyurethanes, to improve resource utilization, and to reduce environmental pollution. On the other hand, phenolic compounds and amine compounds can be introduced to effectively slow down the oxidation reaction of a material and improve its oxidation resistance.

## Author contributions

Writing – original draft preparation, writing – review and editing, Y. M.; writing – original draft preparation, X. Y. H. and M. Y.; conceptualization, writing – review and editing, supervision, funding acquisition, C. Q. H. and L. X. H. All authors have read and agreed to the published version of the manuscript.

## Conflicts of interest

The authors declare no conflicts of interest.

## Supplementary Material
